# First case report of cutaneous sporotrichosis (*Sporothrix* species) in a cat in the UK

**DOI:** 10.1177/2055116920906001

**Published:** 2020-02-14

**Authors:** Nikoleta Makri, Gavin K Paterson, Fiona Gregge, Catriona Urquhart, Tim Nuttall

**Affiliations:** 1 Dermatology Service, Hospital for Small Animals, University of Edinburgh, Royal (Dick) School of Veterinary Studies, Roslin, UK; 2 Easter Bush Pathology, University of Edinburgh, Royal (Dick) School of Veterinary Studies, Roslin, UK; 3 Aberdeen PDSA Pet Hospital, Aberdeen, UK; 4 Abervet (Nigg Kirk Hall Surgery), Aberdeen, UK

**Keywords:** Cutaneous mycosis, molecular identification, *Sporothrix pallida*, *Sporothrix* species, sporotrichosis

## Abstract

**Case summary:**

A 12-year-old female neutered indoor–outdoor domestic longhair cat presented with frequent sneezing and a nodular, suppurative lesion on its dorsal nose. Histopathological examination revealed a fungal granuloma. PCR and sequencing of the ribosomal internal transcribed spacers (ITS) regions (ITS-F and ITS-R) confirmed an infection with a *Sporothrix* species. Further sequencing of the beta-tubulin and calmodulin genes confirmed *Sporothrix humicola*, which lies within the *Sporothrix pallida* complex. The cat had concurrent diabetes mellitus, which responded to insulin therapy and diet. Oral itraconazole at 10 mg/kg PO q24h resulted in resolution of the lesions after 12 months. Treatment was well tolerated.

**Relevance and novel information:**

This is the first report of sporotrichosis in a cat in the UK and only the fifth worldwide involving the *S pallida* complex. Clinicians, pathologists and microbiologists need to be aware of the potential of *Sporothrix* infections in the UK and the ability of *S pallida* complex to cause opportunistic infections. Molecular techniques can achieve rapid and accurate identification of rare fungal organisms. A precise diagnosis with molecular testing can provide information regarding prognosis, treatment and zoonotic implications.

## Introduction

Sporotrichosis is a chronic mycosis caused by fungi of the genus *Sporothrix.*^1,2^ The traditional route of transmission in humans and animals is by traumatic inoculation of contaminated material (eg, soil and decaying organic matter) into subcutaneous tissues.^3^ Alternative routes include direct transmission (cat-to-cat and cat-to-human) through scratching and biting,^4^ and human inhalation of infectious conidia.^3,4^

Sporotrichosis is an emerging zoonotic disease that is most prevalent in Latin America^5^ but has spread worldwide.^3^ Species within the *Sporothrix schenckii* complex (*Sporothrix brasiliensis, Sporothrix globosa, Sporothrix schenckii sensu stricto* and *Sporothrix luriei*) make up the clinical clade of the *Sporothrix* genus that most commonly affects humans and animals.^3,6^ *S brasiliensis* is the main causative agent of feline sporotrichosis. In contrast, the species within the *Sporothrix pallida* complex (*Sporothrix mexicana, Sporothrix chilensis, Sporothrix palmicuminata, Sporothrix humicola, Sporothrix pallida* and *Sporothrix stylites*) are rarely pathogenic and are usually self-resolving. There have only been four published case reports attributed to *S pallida* complex: two in humans,^7,8^ one in a cat and one in two eastern quolls (both recent reports from Australia).^9,10^ In this case report, we describe the first confirmed case of feline sporotrichosis in the UK. This was attributed to a species within the *S pallida* complex.

## Case description

**Figure 1:**
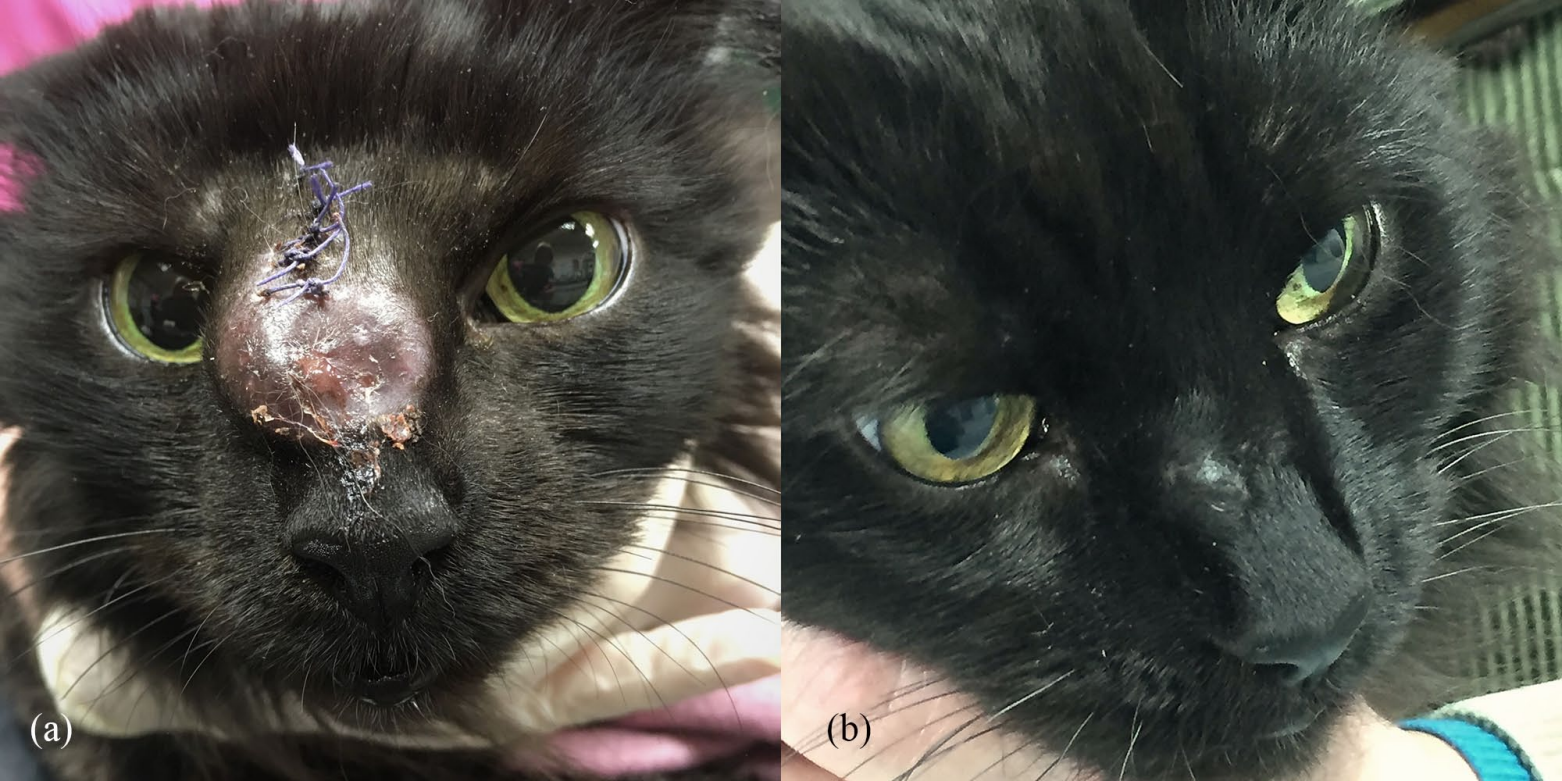
(a) The cat shortly after presentation with a large suppurative nodule on its dorsal nose (the sutures were used to close the incisional biopsy site). (b) Complete resolution of the cutaneous lesions with only residual scarring at the biopsy site after 12 months of itraconazole treatment (10 mg/kg PO q24h)

A 12-year-old client-owned indoor–outdoor, vaccinated, female neutered domestic longhair cat was initially presented with a history of a nodular lesion on its dorsal nose, frequent sneezing and intermittent bilateral serosanguinous nasal discharge. The nodule was hard on palpation, non-ulcerative and non-painful (Figure 1). According to the owner, this had developed acutely. There was no history of previous injury and general physical examination (including regional lymph node palpation) was unremarkable. Initially, the cat was treated empirically by the referring veterinarian with amoxicillin–clavulanate (25 mg/kg PO q12h) and prednisolone (0.8 mg/kg PO q24h), but there was no significant improvement of the clinical signs. Thus, haematology and routine serum biochemistry were performed, which showed increased alanine aminotransferase (139 U/l; reference interval [RI] 18–77 U/l) and alkaline phosphatase (91 U/l; RI 11–67 U/l) with hyperglycaemia (21.1 mmol/l; RI 3.80–7.6 mmol/l). Further serum fructosamine testing (597 µmol/l; RI: 221–341 µmol/l) confirmed a diagnosis of diabetes mellitus and the cat was started on subcutaneous insulin therapy (Caninsulin 40 IU/ml initially started at 2 IU q12h and finally stabilised at 3.5 IU q12h). Thoracic and head radiographs showed soft tissue swelling without bone lysis on the nasal region, but there were no abnormalities detected in the chest.

**Figure 2:**
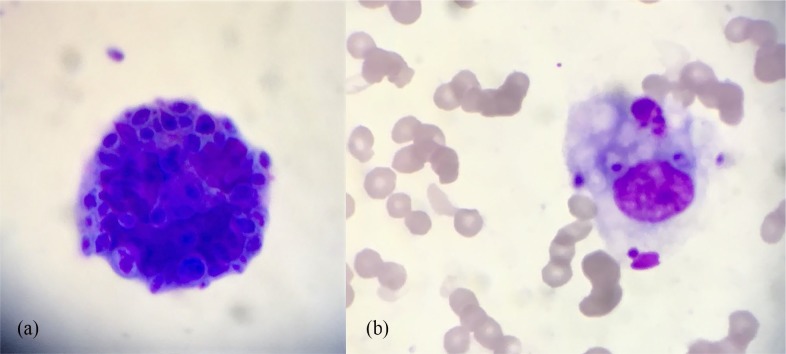
Indirect impression smear cytology of the cutaneous lesion showing oval encapsulated bodies (a), some of which have been phagocytosed by macrophages (b) (Rapi-Diff II stain, × 400 magnification)

Fresh nasal discharge was collected for cytological, bacterial and mycological analysis. The lesion was surgically debulked and nasal tissue was submitted for histopathology. Postoperative analgesia was achieved with meloxicam 0.05 mg/kg PO q24h. Cytology revealed pyogranulomatous inflammation with numerous basophilic round-to-oval yeasts surrounded by a thin clear halo (Figure 2). Histopathology of the skin revealed diffuse infiltration of macrophages together with smaller numbers of neutrophils, lymphocytes and plasma cells. Spherical-to-oval fungal elements were observed within macrophages (confirmed with periodic acid–Schiff staining). Bacterial culture was negative.

A white mould-like growth was seen after overnight incubation at room temperature and at 37^o^C on Sabouraud dextrose agar with chloramphenicol. Long, delicate conidiophores were seen on microscopy. The isolate was classified phenotypically as a *Trichosporon* species using the YST identification card on an automated VITEK 2 analyser (Biomerieux). However, sequencing of the ribosomal internal transcribed spacers (ITS) with the primers ITS-1 and ITS-4, identified the isolate as being a *Sporothrix* species.^11^ Sequencing of the calmodulin and beta-tubulin genes using primer pairs CAL-Fw and CAL-Rv and Bt2a and Bt2b, respectively, was used to further identify the isolate.^12,13^ Using these partial gene sequences, the best matches in NCBI Blast searches limited to type material were to *S humicola* in both cases. The partial calmodulin gene sequence matched 100% to that of *S humicola* CBS 118129 (corresponding to nucleotide positions 1–564 of Genbank accession KX590808.1) and the partial beta-tubulin gene sequence matched 98.2% with that of *S humicola* CMW7618 (=CBS 118129, corresponding to nucleotide positions 1–292 of Genbank accession EF139100.1).

Treatment with itraconazole (10 mg/kg PO q24h) was started. The lesion gradually regressed and was completely resolved with only some scar tissue present at the biopsy site after 12 months of treatment (Figure 1). The itraconazole was well tolerated by the cat, with no reported side effects. The diabetes mellitus remained stable with low-calorie/high-fibre diets and insulin therapy.

## Discussion

This is the first confirmed case of feline sporotrichosis in the UK. It was attributed to *S humicola*, which is a species within the *S pallida* complex. *Sporothrix* species can cause cutaneous and disseminated mycoses in humans, cats and other animals, although most infections are associated with species in the *S schenckii* complex.^3^ *S brasiliensis* accounts for most feline cases and epidemic outbreaks have been reported.^3,14^ Studies in experimental mouse models, in contrast, show that the *S pallida* complex is much less pathogenic.^15^ Naturally occurring infections are rare and sporadic.^3^ The affected cat had typical clinical signs of localised nodular and ulcerative fungal infection of the face, with no evidence of disseminated disease. It is therefore likely that the infection was associated with a penetrating trauma or a cat scratch.

The clinical severity of sporotrichosis varies from asymptomatic and self-resolving to extensive and disseminated, depending on the route of transmission, the *Sporothrix* species and the host immune response.^3^ Most feline lesions are limited to areas that contact soil and/or are common sites of injury such as the nose, ears and digits.^3^ In contrast to humans, lymphatic involvement is rare in cats,^16^ but unusual presentations such as otitis externa have been described in cats and dogs.^17–19^

This cat’s underlying diabetes mellitus is likely to have made it more susceptible to infection. The cat had not been tested for other causes of being immunocompromised (owing to owner’s considerable financial constraints), such as feline leukaemia virus or feline immunodeficiency virus, and these cannot be entirely ruled out. Opportunistic fungal pathogens have to overcome the host’s immune response to cause disease, and immunocompromised cats are more susceptible.^3^ Identifying and managing underlying conditions is therefore important.

A rapid and accurate diagnosis of an opportunistic fungal infection can be challenging. Cytology is a quick and easy test; as in this case, it should reveal pyogranulomatous inflammation.^3^ Fungal elements may be visible, but their absence cannot be used to rule out a fungal infection and, conversely, their presence may simply reflect environmental contamination.^20,21^ Histopathology typically confirms pyogranulomatous inflammation.^22^ Round-to-oval yeasts and hyphae are seen in most cases, especially if fungal-specific stains are used, but cases of sporotrichosis without histopathological evidence of the fungi have been reported.^23^ In addition, the cytological and histopathological appearance of *Sporothrix* species can be variable and resemble other fungal organisms.^20–22^ Further diagnostics are therefore required for a definitive diagnosis.

Fungal culture is the gold standard for an accurate diagnosis of sporotrichosis.^5^ However, this relies on clinicians considering fungal infection as a differential diagnosis for these lesions and submitting material for fungal culture. In addition, it can take up to 2–4 weeks to report samples as negative. Finally, relying on morphological characteristics of the fungal elements and biochemical tests may, as in this case, incorrectly identify the organism.

Molecular methods offer a precise diagnosis for fungal infections. This aids appropriate treatment choices, prognostic predictions and zoonotic risk assessments. In this case, a sequential approach after initial fungal culture was used. PCR and sequencing of the ribosomal ITS regions is the standard approach to fungal identification. This confirmed an infection with a *Sporothrix* species but did not allow confident speciation. Further sequencing of the beta-tubulin and calmodulin genes confirmed that the isolate lay within the *S pallida* complex and identified it as *S humicola.*^24^ A more rapid diagnosis could be achieved by applying these molecular techniques directly to fresh tissue. However, these sensitive techniques can amplify contaminants and other tests (eg, cytology and histopathology) should also be used to confirm the infection.

Treatment of sporotrichosis can be challenging. The traditional treatment in cats is oral administration of an imidazole or triazole antifungal, with or without potassium iodide.^3,25,26^ Continuation of treatment for at least 30 days after clinical cure is essential to reduce the chances of recurrence. Itraconazole and potassium iodine are commonly used, although potassium iodide has a high incidence of side effects in cats (particularly iodism).

The prognosis will depend on diagnosis and management of any underlying problems.^10,26^ In this case, accurate identification of infection with an *S pallida* complex isolate of low pathogenicity and zoonotic risk suggested a fair prognosis with itraconazole treatment. In addition, the good response of the diabetes mellitus to insulin therapy contributed to the successful resolution. It is difficult to determine the relative roles of the antifungal treatment and management of the diabetes mellitus in this case. It is possible that the infection could have resolved with management of the diabetes mellitus alone, but treatment of opportunist fungal infections is recommended.^27^ The prolonged course of itraconazole was well tolerated, although this can be associated with gastrointestinal disease and hepatopathy, and thus liver enzymes should be regularly monitored.^3,26^

## Conclusions

*Sporothrix* species are found worldwide, although infections are most common in subtropical and tropical climates. To our knowledge, this is the first confirmed case of feline sporotrichosis in UK and only the fifth report worldwide of an infection with the *S pallida* complex, which is considered a low pathogenic clade of *Sporothrix* species. This should therefore be considered as a cause of nodular and other pyogranulomatous lesions, especially in immunocompromised individuals. Molecular techniques can be used to rapidly and accurately identify fungal agents, facilitating treatment, prognosis and zoonotic considerations.
